# Delayed Gastric Emptying and Gastric Remnant Function After Pancreaticoduodenectomy: A Systematic Review of Objective Assessment Modalities

**DOI:** 10.1007/s00268-022-06784-7

**Published:** 2022-10-23

**Authors:** Tim H.-H. Wang, Anthony Y. Lin, Keno Mentor, Gregory O’Grady, Sanjay Pandanaboyana

**Affiliations:** 1 Department of Surgery, University of Auckland, Auckland, New Zealand; 2 Auckland Bioengineering Institute, University of Auckland, Auckland, New Zealand; 3 HPB and Transplant Unit, Freeman Hospital, Newcastle, UK; 4 Population Health Sciences Institute, Newcastle University, Newcastle, UK

## Abstract

**Background:**

Delayed gastric emptying (DGE) is a frequent complication after pancreaticoduodenectomy (PD). The diagnosis of DGE is based on International Study Group for Pancreatic Surgery (ISGPS) clinical criteria and objective assessments of DGE are infrequently used. The present literature review aimed to identify objective measures of DGE following PD and determine whether these measures correlate with the clinical definition of DGE.

**Methods:**

A systematic search was performed using the MEDLINE Ovid, EMBASE, Google Scholar and CINAHL databases for studies including pancreatic surgery, delayed gastric emptying and gastric motility until June 2022. The primary outcome was modalities undertaken for the objective measurement of DGE following PD and correlation between objective measurements and clinical diagnosis of DGE. Relevant risk of bias analysis was performed.

**Results:**

The search revealed 4881 records, of which 46 studies were included in the final analysis. There were four objective modalities of DGE assessment including gastric scintigraphy (*n* = 28), acetaminophen/paracetamol absorption test (*n* = 10), fluoroscopy (*n* = 6) and the ^13^C-acetate breath test (*n* = 3). Protocols were inconsistent, and reported correlations between clinical and objective measures of DGE were variable; however, amongst these measures, at least one study directly or indirectly inferred a correlation, with the greatest evidence accumulated for gastric scintigraphy.

**Conclusion:**

Several objective modalities to assess DGE following PD have been identified and evaluated, however are infrequently used. Substantial variability exists in the literature regarding indications and interpretation of these tests, and there is a need for a real-time objective modality which correlates with ISGPS DGE definition after PD.

## Introduction

Delayed gastric emptying (DGE) is one of the most common complications following pancreaticoduodenectomy (PD), with postoperative DGE rates ranging between 10 and 45% [1, 2]. DGE can significantly increase postoperative morbidity, prolong hospital stay and increase healthcare costs [3, 4].

Historically, there have been several definitions of DGE, with studies using different definitions leading to significant challenges in interpreting findings across studies. In 2007, the International Study Group for Pancreatic Surgery (ISGPS) consensus statement standardized the definition of DGE [4]. While allowing for a standardized measure of DGE, this definition is reliant on subjective clinical judgement based on the duration of nasogastric (NG) tube intubation and reinsertion. The DGE grade can also only be established at the end of the patient's clinical course. Nevertheless, there have been several publications validating the ISGPS definition of DGE [1, 5, 6]. Furthermore, DGE can be classified into those relating to the surgical procedure itself (primary DGE) or to postoperative complications, e.g., pancreatic fistulas, hemorrhages or intra-abdominal abscess (secondary DGE) [7]. A more objective measure of DGE after PD may allow a real-time and impartial assessment to guide clinical management and develop strategies to prevent or treat DGE.

The aim of this systematic review was to identify the objective assessment modalities of DGE used in the literature following PD. This study also aimed to identify correlations between current clinical definitions of DGE and objective DGE assessments, along with correlations between postoperative symptoms and the objective assessment of DGE.

## Materials and methods

This systematic review was completed in accordance with the PRISMA 2020 statement [8] and was prospectively registered with PROSPERO (ID: CRD42021260141).

### Literature search

A systematic literature review of MEDLINE (OVID) (1946-June 2022), EMBASE (1980-June 2022), Google Scholar and CINAHL (1982-June 2022) databases was performed in June 2022.

In brief, the search was conducted using the following Medical Subject Heading (MeSH) terms and text words: “pancreaticoduodenectomy”, “pancreatectomy”, “Whipples” AND “gastroparesis”, “postgastrectomy syndrome”, “gastric emptying”, “delayed gastric emptying”, “DGE”, “gastrointestinal motility”, “gastrointestinal transit”, limiting to human studies in English. Reference lists of relevant records were also manually searched for additional eligible publications.

### Inclusion and exclusion criteria

This literature search included studies involving pancreaticoduodenectomy (classical, pylorus-preserving or other variations) and excluded other forms of pancreatic resections such as distal, total and central pancreatectomy. The search only included studies that assessed DGE using a non-clinical and objective measure. Meta-analyses, review articles, case reports (with *n* ≤ 5), letters to the editors, conference proceedings and abstracts were excluded.

### Data extraction

Two independent reviewers (THHW, AL) screened and assessed each article for inclusion and extracted data. A title and abstract screen were first performed, followed by a full-text review. Discrepancies were resolved by the senior author (SP). Data on the type of objective measure of DGE, how it was performed, whether there was any correlation between clinical and objective measures of DGE or between symptoms (not otherwise included in the clinical DGE definitions) and the objective measure of DGE were extracted. Data on routine exclusion of mechanical obstruction at the gastrojejunal anastomosis, whether primary and secondary DGE was clearly differentiated and whether the objective DGE results altered management were also extracted. Three risk of bias tools were used, including the revised Cochrane risk-of-bias tool for randomized trials (ROB 2), the Newcastle–Ottawa Scale (NOS) for cohort studies, and the Methodological Index for Non-Randomized Studies (MINORS) for case–control and case series [9–11].

## Results

### Included studies

**Fig. 1:**
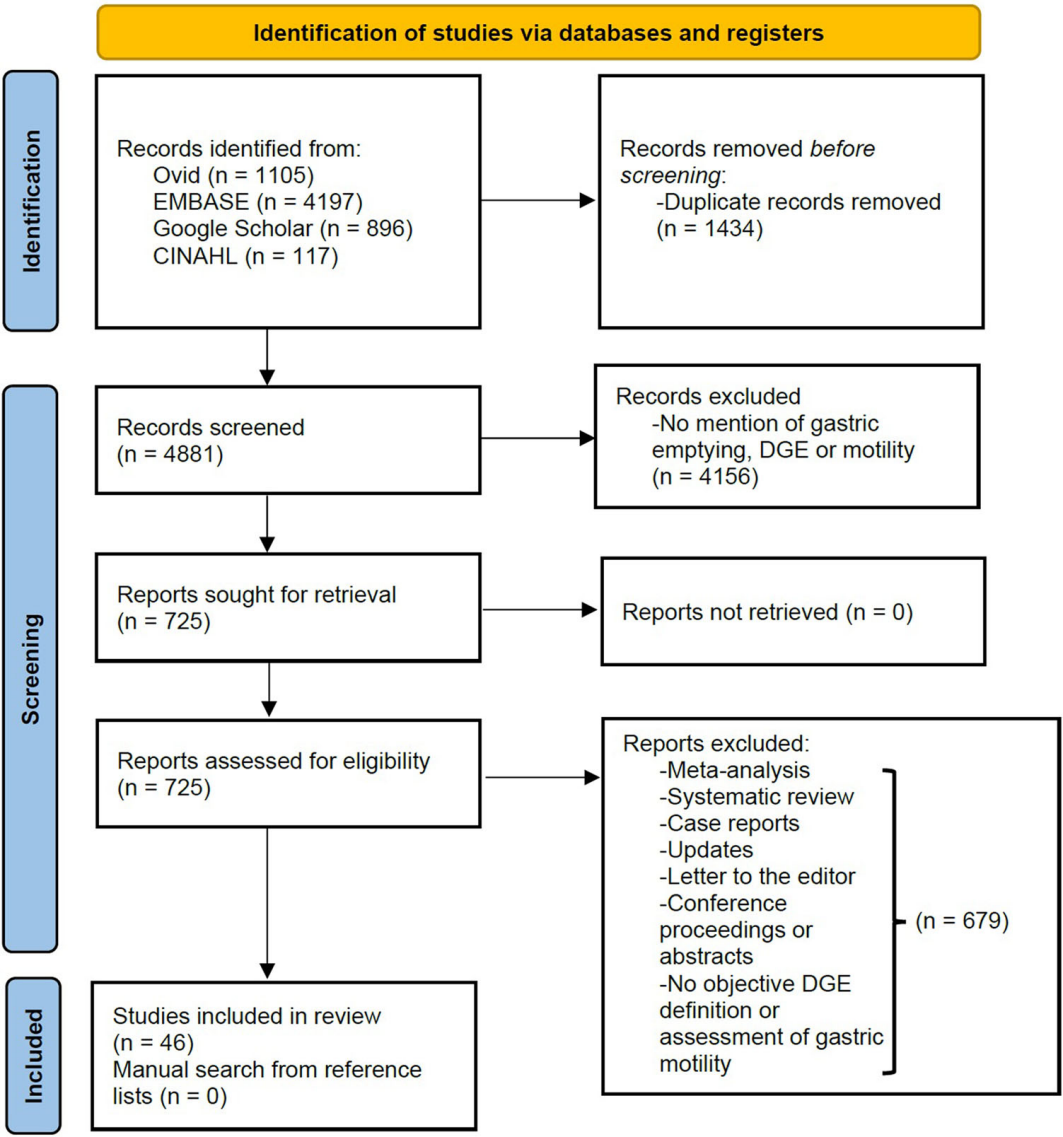
PRISMA 2020 flow diagram of the included records

In total, 4881 articles were identified from the initial search, of which 46 articles met the inclusion criteria and formed the basis of the systematic review (PRISMA diagram shown in Fig. 1). There were 7 randomized control trials (RCT), 16 cohort studies, 8 case–control studies and 15 case series. These studies encompassed 4 different objective measures of DGE: 28 studies used gastric scintigraphy (Table 1), 10 studies used acetaminophen/paracetamol absorption test, 6 studies used fluoroscopy, and 3 studies used ^13^C-acetate breath test (Table 2). One study used both the acetaminophen/paracetamol absorption and fluoroscopy in the early and late postoperative phase, respectively [12]. Only one study specified the routine exclusion of mechanical obstruction as a cause of DGE [13], though several studies performed endoscopy or barium radiography but not specifically to assess for mechanical obstruction. No study clearly specified a subgroup analysis on primary or secondary DGE, however, several studies compared the rates of postoperative complications in the DGE and non-DGE cohorts [14–20]. No study used the results of the objective measure of DGE to alter patient management. Relevant results are presented in Tables 1 and 2.

**Table 1:** Characteristics of studies using gastric scintigraphy

Year	Author	Study design	Patient number	Method of assessing DGE	Time	DGE definition (s)	Results	Routine exclusion of mechanical obstruction clearly defined	Primary and secondary DGE clearly differentiated	Was results of the objective assessment of DGE used to alter treatment?	Risk of bias score
*Gastric scintigraphy*											
1986	Braasch et al. [21]	Cohort	71 Hemi-pancreatectomy (PD) 13 Total pancreatectomy 3 Completion total pancreatectomy 5 Controls	•Early post-op: Length of time with NG tube •Late post-op: ^113m^In labelled liquid or ^99m^Tc labelled solid meal. Radioactivity was recorded by scintigraphy	•Post-op • ≥ 4 months	•Early post-op: NG intubation for > 7 days •Late post-op: Not defined. Statistical significance compared to controls	•6 patients had biliary fistula •7 patients had pancreatic fistulas or collections	No	No	No	7
1987	Patti et al. [22]	Case–control	10 PPPD 5 Controls	•^99m^Tc labelled meal. Radioactivity was recorded by scintigraphy at regular intervals	•1–45 months	•Abnormal if 2 or more points were more than 2 standard deviations from the mean of the control subjects OR more than 20% of activity remains in the stomach after 3 h	Objective gastric emptying results •6 had normal gastric emptying •3 had rapid gastric emptying •1 had delayed gastric emptying •No mention of fistula or leak rates	No, but endoscopic assessment performed on a single patient	No	No	16
1988	Fink et al. [13]	Case–control	6 PD 6 PPPD	•^99m^Tc labelled meal (solid and liquid phase). Radioactivity was recorded by scintigraphy at regular intervals	•1–7 years	•Statistical significance compared to the different groups	•Liquid phase-emptying took longer in PD than PPPD. PPPD similar to controls •Solid phase- no difference between groups	Yes	No	No	18
1989	Hunt and McLean [23]	Case series	11 Head of pancreas resection 5 Total pancreatectomy	•Early post-op: Length of time with NG tube •Late post-op: Liquid emptying and solid emptying was completed using "dual isotope technique" (not specified)	•Post-op •3 months	•Early post-op: Not defined •Late post-op: Abnormal if outside the criteria of 50% liquid emptying between 15–20 min OR 0.8–1.0% per minute clearance of solid emptying	•Liquid gastric emptying- 2 rapid emptying, 6 normal gastric emptying, 1 delayed gastric emptying •Solid gastric emptying- 2 rapid emptying, 5 normal gastric emptying, 2 delayed gastric emptying •Pancreatic fistula associated with rapid gastric emptying in 1, pancreatic leak associated with delayed gastric emptying in 1 •No real relationship between early subjective DGE and late gastric emptying status	No, but implied	No, but intra-abdominal complications recorded	No	13
1991	Lerut et al. [24]	Case series	18 Partial pancreatectomy 2 Total pancreatectomy	•Early post-op: Length of time with NG tube •Late post-op: ^99m^Tc labelled meal (solid and liquid phase). Radioactivity was recorded by scintigraphy at regular intervals	•Post-op •3–6 months	•Early post-op: NG tube requirement for > 7 days •Late post-op: not defined	•Patients with pancreatic fistula required a longer post-op NG drainage (number not specified) •Gastric emptying rapid in 1, normal in 9, delayed in 2 •Pancreatic fistula associated with 1 rapid emptying and 1 delayed emptying	No	No, but intra-abdominal complications recorded	No	11
1993	De Bernardinis et al. [25]	Case series	11 PPPD with modifications	•Early post-op: Length of time with NG tube •Late post-op: ^99m^Tc labelled meal (solid phase). Radioactivity was recorded by scintigraphy at regular intervals	•6 months	•Early post-op: NG tube intubation for > 10 days •Late post-op: T_1/2_ was longer than 80 min	•Early post-op: 4 patients had 'gastric stasis' •Late post-op: 2 patients had delayed gastric emptying	No. Endoscopy performed, but not for assessment for mechanical obstruction	No	No	13
1993	Kingsnorth et al. [26]	Case series	30 PPPD	•Early post-op: Length of time with NG tube, passage of flatus, oral tolerance •Late post-op: ^99m^Tc labelled solid meal. Radioactivity was recorded by scintigraphy	•Post-op •3–18 months	•Early post-op: NG intubation for > 7 days and commencement of oral fluids after 7 days •Late post-op: Normal defined as > 50% emptying after 60 min in controls	•Early post-op: 1 patient (of 30) had delayed emptying •Late post-op: All normal	No. Endoscopy performed, but not clearly defined to assess for mechanical obstruction	No	No	13
1993	Williamson et al. [27]	Cohort	12 PD 24 PPPD	•Early post-op: Length of time with NG tube/oral tolerance •Late post-op: ^113m^In labelled liquid and ^99m^Tc labelled solid meal. Radioactivity was recorded by scintigraphy	•Post-op •2 months–5 years	•Early post-op: NG intubation for > 8 days •Late post-op: (a) Upper limit of normal t_1/2_ for liquid emptying- 70 min (b) Upper limit of normal t_1/2_ for solid emptying- 110 min	•Early post-op: delayed in 2/24 of the PPPD patients, 1/12 in PD (had anastomotic dehiscence) •Late post-op: (a) 57% in PPPD and 45% in PD normal. (13/23, 5/11) (b) 42% in PPPD and 17% in PD normal. (10/24,2/12)	No. Endoscopic and barium studies were mentioned in the results to assess for mechanical obstruction in some patients	No	No	7
1993	Yeo et al. [28]	RCT	18 PD 100 PPPD	•Length of time with NG tube, oral tolerance •^113m^In labelled liquid and ^99m^Tc labelled solid meal. Radioactivity was recorded by scintigraphy	•Post-op •10 days	•NG intubation for > 10 days and (1) one of the following: (a) emesis after nasogastric tube removed (b) postoperative use of prokinetic agents after postoperative day 10 (c) reinsertion of nasogastric tube (d) failure to progress with diet Or (2) nasogastric tube in place fewer than 10 days plus two of (a)–(d) above •Statistical significance compared to the different groups	•Erythromycin group (11/58), Control group (18/60) had DGE OR Erythromycin group (7/49), Control group (14/47) had DGE excluding complications •Erythromycin associated with improved gastric emptying	No	No	No	Low
1993	Yung et al. [29]	Case series	50 PPPD 4 PD	•^113m^In labelled liquid and ^99m^Tc labelled solid meal. Radioactivity was recorded by scintigraphy	•7–15 days	•Statistical significance compared to the different views	•No statistical difference between the 3 different views	No	No	No	19
1995	Pastorino et al. [30]	Case series	15 PPPD	•^99m^Tc labelled solid meal. Radioactivity was recorded by scintigraphy	•3–30 months	•Normal range of T_1/2_ was defined as between 40 and 70 min	•Earlier follow-up was associated with higher half-life than later follow-up	No. Endoscopy performed, but not for assessment for mechanical obstruction	No	No	13
1997	Bruno et al. [31]	Case–control	7 PD 5 PPPD	•^99m^Tc labelled solid meal and ^170^Er labelled ECPM. Radioactivity was recorded by scintigraphy	•30 ± 12 months	•Statistical significance compared to the different groups	•Transit time between ECPM vs pancake meal were not statistically different •In PPPD, transit of ECPM was delayed compared to pancake meal	No	No	No	18
1998	Lupo et al. [32]	Case series	17 PPPD	•Early post-op: NG tube output •Late post-op: ^99m^Tc labelled solid meal. Radioactivity was recorded by scintigraphy	•Post-op •5–7 months	•Early post-op: NG output for > 1L/day for > 7 days •Late post-op: DGE was defined as T_1/2_ > 85 min	•Early post-op: 1/17 had DGE •Late post-op: Rapid gastric emptying 3/11, normal 5/11, delayed gastric emptying 3/11	No	No	No	18
1999	Hishinuma et al. [33]	Case–control	24 PPPD 2 Controls	•^113m^In labelled meal with ^99m^Tc intravenous injection. Radioactivity was recorded by scintigraphy	•28 days to 67 months	•Not defined but the 2 normal controls had 43% and 66% gastric retention at 1 h after imaging	•Rate of gastric emptying improved compared to pre- and post- 2 months post-op	No	No	No	16
1999	Sumida et al. [34]	Cohort	14 PPPD with preserved superior pyloric branch 13 PPPD without nerve preservation	•^99m^Tc labelled meal. Radioactivity was recorded by scintigraphy	•1 month	•Statistical significance compared to the different groups	•3/11 showed delayed gastric emptying	No	No	No	7
1999	Thor et al. [35]	Cohort	18 PD 10 PPPD	•Liquid gastric emptying: Ultrasound used (not specified) •Solid gastric emptying: ^99m^Tc labelled solid meal. Radioactivity was recorded by scintigraphy •Electrogastrography performed	•pre-op and 2 months	•Normal range of T_1/2_ was 45 ± 9 min and delayed if t_1/2_ was increased more than 1 standard deviation above normal •EGG- not defined	•Pre-op: (a) Liquid DGE in 5/18 and 2/10 (b) Solid DGE in 8/18 and 2/10 •Post-op (a) Liquid rapid GE in 16/18 and 0/10 (b) Liquid DGE in 1/18 and 6/10 (c) Solid rapid GE in 12/18 and 0/10 (d) Solid DGE in 4/18 and 5/10	No	No	No	7
2000	Sato et al. [36]	Case series	8 PD 8 PPPD	•^99m^Tc labelled meal. Radioactivity was recorded by scintigraphy	• > 2 years	•Not defined	•Gastric emptying half-life ranged between 9 and 147 min	No	No	No	12
2000	Sato et al. [37]	Cohort	9 PD- Imanaga 9 PPPD- Imanaga	•^99m^Tc labelled meal. Radioactivity was recorded by scintigraphy	• > 2 years	•Statistical significance compared to the different groups	•Gastric emptying half-life was significantly shorter in the PDI vs the PpPDI group in a sitting position but no difference in the supine position	No	No	No	8
2003	Caronna et al. [38]	Case series	25 PD	•Scintigraphy (not specified) was completed	•3 months	•Not defined	•Good rhythmic and regular gastric emptying	No. Endoscopy performed, but not for assessment for mechanical obstruction	No	No	10
2005	Kim et al. [39]	Case series	47 PPPD	•Clinical: Inability to tolerate oral diet •Objective: ^99m^Tc labelled meal. Radioactivity was recorded by scintigraphy	•Post-op •Pre-op and post-op	•Clinical: Inability to tolerate oral diet by 8 days post-op •Objective: DGE defined as gastric retention of the test meal > 55% at 2 h	• Preoperative GET: abnormal in 20/39 •Postoperative GET: abnormal in 13/35	No	No	No	20
2005	Shan et al. [40]	RCT	23 PPPD	•Clinical: Length of time with NG tube •Objective: ^99m^Tc labelled meal (solid and liquid phase). Radioactivity was recorded by scintigraphy	•Post-op •14 days	•Clinical: Not defined •Objective: oDGE defined as gastric emptying T_1/2_ increased more than the mean ± 2SD (58.2) mins	•Subjective: sDGE was higher in the somatostatin group (9/11, 82%) vs the non-somatostatin group (3/12, 25%) •Objective: oDGE was higher in the somatostatin group (10/11, 91%) vs the non-somatostatin group (3/12, 25%)	No	No	No	Low
2005	Shan et al. [41]	Cohort	33 PD 21 PPPD	•Clinical: Length of time with NG tube •Objective: ^99m^Tc labelled meal (solid and liquid phase). Radioactivity was recorded by scintigraphy	•Post-op •Pre-op, 14 days and 6 months	•Clinical: NG intubation for ≥ 10 days •Objective: (a) Delayed liquid emptying diagnosed if T_1/2_ > 23 min (b) Delayed solid emptying diagnosed if T_1/2_ increased by more than 2 standard deviations above the mean	•Subjective: sDGE higher in PPPD than PD (9/21 vs 5/33) at 14 days and 0 for both groups at 6 months •Objective: (a) Pre-op- PPPD group (5/21 had delayed LGE, 6/21 had delayed SGE), PD group (3/33 had delayed LGE, 7/33 had delayed SGE) (b) Post-op day 14- PPPD group (76% LGE, 42% SGE), PD group (91% LGE, 88% SGE) (c) 6 months- PPPD group (4.7% LGE, 4.7% SGE), PD group (37% LGE, 30% SGE)	No	No	No	7
2007	Shan et al. [42]	Cohort	21 PPPD 20 Controls	•Clinical: Length of time with NG tube •Objective: ^99m^Tc labelled meal (solid and liquid phase). Radioactivity was recorded by scintigraphy	•Post-op •12 days (liquid) and 14 days (solid) and 6 months	•Clinical: NG intubation for ≥ 10 days OR if patient experiences emesis after removal of NG tube, reinsertion of NG tube or failure to progress with diet •Objective: (a) Delayed liquid emptying diagnosed if T_1/2_ > 23 min (b) Delayed solid emptying diagnosed if T_1/2_ increased by more than 2 standard deviations above the mean (c) Proximal to distal stomach radiation count (P/DR) ratio was used	•Subjective: 9/21 had sDGE •Objective: P/DR for the patients were lower than the controls for both liquid and solid phase at both 14 days and at 6 months in general	No	No	No	5
2008	Kollmar et al. [14]	RCT	62 PPPD 5 PD	•Length of time with NG tube •^99m^Tc labelled meal. Radioactivity was recorded by scintigraphy	•Post-op •7 days	•NG intubation for > 10 days and/or intolerance of normal diet beyond 14 days •Not defined	•Subjective: 7/35 in octreotide group 6/32 in control group •Objective: 12/35 in octreotide group 10/32 in control group for GE half-life > 60 min	No	No, but pancreatic fistula was analysed for DGE vs non-DGE	No	Low
2013	van Samkar et al. [43]	Case–control	28 PPPD 16 Double-bypass procedure	•Clinical: ISGPS consensus •Objective: ^99m^Tc labelled meal (solid phase). Radioactivity was recorded by scintigraphy	•Post-op •Pre-op and 7 days	•Clinical: ISGPS criteria for DGE (B or C) •Objective: Upper normal limit of normal is retention of 60% at 2 h	•Subjective: 12/44 had DGE •Objective: 19/44 had objective DGE •Postoperative scintigraphy is associated with severity of subjective DGE	No	No	No	19
2015	Eshuis et al. [44]	RCT	38 retrocolic 35 antecolic 63 PPPD 10 PD	•Clinical: ISGPS consensus •Objective: ^99m^Tc labelled meal (solid phase). Radioactivity was recorded by scintigraphy	•Post-op •Pre-op and 7 days	•Clinical: ISGPS criteria for DGE (B or C) •Objective: Upper normal limit of normal is retention of 60% at 2 h	•Subjective: (a) Antecolic (14/35) Retrocolic (13/38) had DGE (b) Antecolic (3/35) Retrocolic (7/38) had DGE excluding complications •Objective: (a) All patients normal pre-op (b) Antecolic group (7/20) (c) Retrocolic group (12/23) had DGE at 7 days post-op	No	No	No	Low
2017	Samaddar et al. [15]	Case–control	21 PD	•Clinical: ISGPS consensus •Objective: ^99m^Tc labelled meal (solid phase). Radioactivity was recorded by using a SPECT machine	•Post-op •Pre-op and 10 and 21 days	•Clinical: ISGPS criteria for DGE (A or above) •Objective: Normal taken as > 50% clearance at 1 h and t_1/2_ of < 80 min	•Subjective: 13/21 had DGE •Objective: (a) POD10- 8/21 had DGE (b) POD21- 5/21 had DGE	No	No, but pancreatic fistula and intra-abdominal infection were analysed for DGE vs non-DGE	No	19
2018	Shahbazov et al. [45]	Case–control	17 PD 66 PPPD	•Clinical: Based on symptoms (not specified) •Objective: Nuclear medicine gastric emptying study (not specified)	•Post-op	•Clinical: Not defined •Objective: Not defined	•15/83 had DGE (unclear which definition was used)	No. Endoscopy performed, but not clearly defined to assess for mechanical obstruction	No	No	18

*AUC* area under the curve, *DGE* delayed gastric emptying, *ECPM* enteric-coated pancreatin microspheres, *GET* gastric emptying time, *ISGPS* International Study Group in Pancreatic Surgery, *LGE* liquid gastric emptying, *NG* nasogastric, *PD* pancreaticoduodenectomy, *PPPD* pylorus-preserving pancreaticoduodenectomy, *RCT* randomized control trial, *SGE* solid gastric emptying, *SPECT* single photon emission computed tomography

**Table 2:** Characteristics of studies using other objective DGE measures

Year	Author	Study design	Patient number	Method of assessing DGE	Time	DGE definition (s)	Results	Routine exclusion of mechanical obstruction clearly defined	Primary and secondary DGE clearly differentiated	Was results of the objective assessment of DGE used to alter treatment?	Risk of bias score
*Acetaminophen/paracetamol absorption test*											
1992	Watanabe et al. [12]	Cohort	10 PD with either Billroth I or Billroth II reconstruction 8 Controls	•Early post-op, subjective: Length of time with NG tube, oral tolerance •Early post-op, objective: Barium fluoroscopy •Late post-op: Acetaminophen added to a meal. Blood tests for acetaminophen levels obtained pre-administrations and serially post-administration	•Post-op • ≥ 3 months	•Early post-op, subjective: Not defined. Time elapsed before removal of intragastric tube and resumption of oral intake •Early post-op, objective: Gastric emptying considered to be adequate according to radiography if the barium ingested (150 mL) was almost entirely eliminated within 1 h •Late post-op: Statistical significance compared to the different groups	•No difference between subjective gastric emptying between 2 reconstruction techniques •Barium fluoroscopy-1/10 had delayed gastric emptying. No difference between the reconstruction techniques •Patients had delayed gastric emptying than controls using the acetaminophen technique	No. Barium radiography was used to assess for gastric emptying, rather than for mechanical obstruction	No	No	6
1995	Ueno et al. [46]	Cohort	8 PD 18 PPPD 8 Cholecystectomy 4 Transabdominal esophageal transection 10 Distal partial gastrectomy 32 Controls	•Acetaminophen added to a meal. Blood tests for acetaminophen levels obtained pre-administrations and serially post-administration	•< 2 months or > 3 months	•Statistical significance compared to the different groups	•Early postoperative period (a) Lower acetaminophen concentration in PPPD and PD compared to controls (b) No difference in acetaminophen concentration in PPPD and PD compared to controls	No	No	No	5
1997	Muller et al. [47]	Cohort	10 Duodenum-preserving pancreatic head resections 10 PPPD 6 Controls	•Acetaminophen added to a meal. Blood tests for acetaminophen levels obtained pre-administrations and serially post-administration	•Pre-op •10 days •6 months	•Statistical significance compared to the different groups	•No clinical DGE •Decreased serum acetaminophen absorption in both groups at 10 days post-op •Increased serum acetaminophen absorption in both groups at 6 months post-op but no different to preoperative findings	No	No	No	7
1998	Kobayashi et al. [48]	Case series	14 PPPD	•Liquid gastric emptying: Acetaminophen added to water. Blood tests for acetaminophen levels obtained pre-administrations and serially post-administration •Solid gastric emptying: Sulphamethizole capsules were given. Blood tests for sulphamethizole levels obtained pre-administrations and serially post-administration	•27–53 days	•Statistical significance compared to the preoperative levels	•Liquid gastric emptying: No significant difference in gastric emptying except for measurement of acetaminophen at 120 min •Solid gastric emptying: Delayed in postoperative period	No	No	No	19
1999	Takeda et al. [49]	RCT	16 PPPD 18 Controls	•Acetaminophen added to a meal. Blood tests for acetaminophen levels obtained pre-administrations and serially post-administration •Electrogastrography performed	•Pre-op and 1, 3, 6, 9, 12 months •2–4 weeks and 6–12 months	•Statistical significance compared to the different time points •EGG- Dysrhythmias were identified	•Gastric emptying was delayed but returned to preoperative levels by 6 months post-op •Cisapride improved gastric emptying early in the post-op period •EGG- dysrhythmias identified	No	No	No	Low
2002	Ohtsuka et al. [50]	Cohort	57 PPPD 25 Controls	•Acetaminophen added to a meal. Blood tests for acetaminophen levels obtained pre-administrations and serially post-administration	•1 month •From 7–14 days and repeated weekly until gastric Phase 3 identified	•Statistical significance compared to the different groups •Time to first gastric phase 3 activity	•Increased delayed gastric emptying compared to preoperative •The mean period before the first appearance of gastric phase 3 was 38 days	No	No	No	6
2005	Strommer et al. [51]	Cohort	18 PD 13 PPPD	•Clinical: Length of time with NG tube •Objective: Paracetamol added to a meal. Blood tests for paracetamol levels obtained pre-administrations and serially post-administration	•Post-op •11 days	•Clinical: NG intubation for ≥ 10 days or recurrent vomiting on day 9–10 post-op •Objective: A delayed gastric emptying rate was defined as T_max_ > 240 min and/or C_max_ < 25 µM	•Subjective: 9/31 had delayed gastric emptying •Objective: 14/28 had delayed gastric emptying (3 excluded) •Lack of correlation between gastric function and objective measures	No	No	No	7
2007	Ohuchida et al. [52]	Cohort	31 PPPD	•Acetaminophen added to a meal. Blood tests for acetaminophen levels obtained pre-administrations and serially post-administration	•1–2 months •6–12 months	•Statistical significance compared to the preoperative levels	•Short-term gastric emptying was slowed •Long-term gastric emptying returned to normal	No	No	No	7
2014	Harmuth et al. [53]	Cohort	13 PD 13 PPPD	•Paracetamol added to a meal. Blood tests for paracetamol levels obtained pre-administrations and serially post-administration	•Between 5–199 months	•Statistical significance compared to the different groups	•PD had better gastric emptying than PPPD	No	No	No	8
2014	Tamandl et al. [16]	RCT	64 PPPD	•Clinical: Length of time with NG tube, oral tolerance •Objective: Paracetamol added to a meal. Blood tests for paracetamol levels obtained pre-administrations and serially post-administration	•Post-op •10 days	•Clinical: NG intubation for > 10 days and one of the following: (a) Emesis after nasogastric tube removed (b) Reinsertion of nasogastric tube (c) Failure to progress with diet (d) Use of prokinetics after day 10 post-op •Objective: Statistical significance compared to the different groups	•Subjective: 6/36 in antecolic group and 6/28 in retrocolic group and DGE •Objective: No difference between antecolic and retrocolic. Patients with clinical DGE had lower paracetamol levels	No	No, but pancreatic fistula and intra-abdominal infection were analysed for DGE vs non-DGE	No	Low
*Fluoroscopy*											
1980	Traverso and Longmire [54]	Case series	8 PPPD	•Barium upper gastrointestinal series •Standard Hunt test- not specified	•2–6 months	•Not defined	•Gastric emptying was normal in all patients •Hunt test normal in 7/8 patients	No	No	No	10
1992	Watanabe et al. [12]	Cohort	10 PD with either Billroth I or Billroth II reconstruction 8 Controls	•Early post-op, subjective: Length of time with NG tube, oral tolerance •Early post-op, objective: Barium fluoroscopy •Late post-op: Acetaminophen added to a meal. Blood tests for acetaminophen levels obtained pre-administrations and serially post-administration	•Post-op • ≥ 3 months	•Early post-op, subjective: Not defined. Time elapsed before removal of intragastric tube and resumption of oral intake •Early post-op, objective: Gastric emptying considered to be adequate according to radiography if the barium ingested (150 mL) was almost entirely eliminated within 1 h •Late post-op: Statistical significance compared to the different groups	•No difference between subjective gastric emptying between 2 reconstruction techniques •Barium fluoroscopy-1/10 had delayed gastric emptying. No difference between the reconstruction techniques •Patients had delayed gastric emptying than controls using the acetaminophen technique	No. Barium radiography was used to assess for gastric emptying, rather than for mechanical obstruction	No	No	6
2001	Abdel-Wahab et al. [17]	Case series	81 PD	•Barium upper gastrointestinal series	•1,3,6,12 months	Not defined	•7/81 patients (8.9%) had delayed gastric emptying	No. Endoscopy performed, but not clearly defined to assess for mechanical obstruction	No, but pancreatic fistula and intra-abdominal infection were analysed for DGE vs non-DGE	No	11
2015	Krishna et al. [18]	Case series	52 PD	•Clinical: Length of time with NG tube, nutritional requirement OR •Objective: Gastrografin^®^ study	•5 days	•DGE defined as (a) Reinsertion of NG after removal (b) Requirement of prolonged TPN or FJ (> 7 days) (c) Hold-up of oral contrast in stomach for more than 4 h after oral Gastrografin® study (Objective)	•DGE present in 3/52 patients	No	No, but postoperative complications were analysed for DGE vs non-DGE	No	14
2018	Nojiri et al. [19]	Case–control	160 subtotal stomach-preserving PD	•Clinical: ISGPS consensus •Objective: Barium meal followed by serial abdominal x-rays	•Post-op •7 days	•Clinical: ISGPS criteria for DGE •Objective: Gastric emptying divided into 3 grades depending on gastric dilation and gastric stasis appearances on imaging	•Subjective: DGE identified in 30 patients. Non-DGE in 130 patients •Objective: 14/64 Grade 2 and 4/64 Grade 3 gastric emptying	No	No, but postoperative complications were analysed for DGE vs non-DGE	No	16
2020	Krishna et al. [55]	Case series	467 PD	•Gastrografin® study	•5 days	•Not defined	•DGE present in 96/467 patients	No. Endoscopy performed, but not for assessment for mechanical obstruction	No	No	12
^*13*^*C-acetate breath test*											
2009	Chijiiwa et al. [56]	RCT	17 Antecolic PPPD 18 Vertical retrocolic PPPD	•Clinical: Length of time with NG tube, oral tolerance •Objective: ^13^C-acetate labelled liquid meal was administered and serial breath samples were taken	•Post-op •Pre-op and 30 days	•Clinical: DGE defined by either (a)NG intubation for > 10 days OR (b) reinsertion of nasogastric tube OR (c) failure to progress with diet by 14 days •Objective: Statistical significance compared to the preoperative levels	•Subjective: DGE found in 1/17 in antecolic group and 4/18 in retrocolic group. Not statistically significant •Objective: Prolonged in both groups. No significant difference	No	No	No	Low
2012	Hiyoshi et al. [57]	Cohort	8 Subtotal stomach-preserving PD 33 PPPD	•^13^C-acetate labelled liquid meal was administered and serial breath samples were taken	•Pre-op and 1,3,6,9,12 months	•Statistical significance compared to the preoperative levels	•Prolonged gastric emptying half-life after 1 month and recovered to postoperative levels after 3–6 months in PPPD •Gastric emptying half-life not statistically different to preoperative levels after 1–3 months but decreased after 6–12 months in SSPPD	No. Assessment for mechanical obstruction mentioned, but not clearly explained	No	No	8
2014	Kawai et al. [20]	Cohort	66 PD 64 PPPD	•^13^C-acetate labelled liquid meal was administered and serial breath samples were taken	•Pre-op and 6,12,24 months	•Statistical significance compared to the different groups	•Time to peak in breath test at 24 months in DGE was delayed compared to non-DGE patients	No	No, but pancreatic fistula and intra-abdominal infection were analysed for DGE vs non-DGE	No	9

*DGE* delayed gastric emptying, *ISGPS* International Study Group in Pancreatic Surgery, *NG* nasogastric, *PD* pancreaticoduodenectomy, *PPPD* pylorus-preserving pancreaticoduodenectomy, *RCT* randomized control trial

### Gastric scintigraphy

28 studies used gastric scintigraphy to diagnose DGE following PD [13–15, 21–45], involving serial imaging to track the transit of isotopes ingested with a meal. Heterogeneous protocols were identified, including the use of different isotopes (^99m^Tc or ^111m^In), test meals, serial imaging time intervals and definitions of DGE. Several studies also differentiated between liquid and solid phase gastric emptying [13, 23, 24, 27–29, 35, 40–42]. Additionally, one study used ^170^Er-labelled enteric-coated pancreatin microspheres along with ^99m^Tc to assess gastric emptying [31]. More recent studies used the standardized technique of gastric scintigraphy based on consensus definition, using a ^99m^Tc-labelled scrambled egg meal to assess solid gastric emptying, followed by serial imaging with a gamma camera at 1, 2 and 4 h following meal ingestion [58]. Residual gastric activity greater than 60% at 2 h was considered DGE [43, 44].

### Acetaminophen/paracetamol absorption test

10 studies used the acetaminophen (also known as paracetamol) absorption test to define DGE following PD. This technique involves ingesting a standard dose of acetaminophen/paracetamol with regular serum acetaminophen/paracetamol concentration monitoring in the subsequent hours [59]. Any elevation in serum concentration indicates the passage of the ‘meal’ out of the stomach, indirectly assessing gastric emptying [53, 60]. Variable dosing was found between studies. Only one study by Strommer et al. [51] defined a numerical threshold for DGE, assessing maximal plasma concentration (< 25 μM) and time to reach this value (> 240 min). No other studies provided a quantitative definition for DGE, rather, they compared the results to different groups within their respective studies or to preoperative results. Additionally, one study used the acetaminophen/paracetamol absorption test for the liquid phase and sulphamethizole capsule for the solid phase [48].

### Fluoroscopy

6 studies used fluoroscopy to define DGE following PD. Following the ingestion of barium or Gastrografin^®^ (sodium amidotrizoate/amidotrizoate meglumine) contrast, serial radiographs were taken to determine the location and amount of contrast to assess gastric retention, emptying and therefore function. The 3 case series identified did not provide a fluoroscopic definition for DGE [17, 54, 55] while 2 studies defined DGE if contrast was present in the stomach after a defined time interval (1 and 4 h, respectively) [12, 18]. Furthermore, Nojiri et al. [19] described a classification system dividing gastric emptying into three grades depending on gastric distension and stasis appearances on fluoroscopic imaging.

### ^13^C-acetate breath test

3 studies used the ^13^C-acetate breath test to define DGE following PD. Following ingestion of a ^13^C-labelled triglyceride meal, serial breath samples were obtained to determine the concentration of exhaled ^13^CO_2_ or other metabolites using spectrometry, and the time to peak ^13^CO_2_ was determined. No quantitative definition for DGE was used, rather, results were compared between different groups within the studies or to preoperative results. All studies were performed preoperatively and at least 1 month after surgery, but not immediately after surgery [61, 62].

### Correlation between clinical and objective DGE

All objective measures of DGE identified in this review had implied or explicit correlations between clinical and objective measures of DGE. These results are summarized in Table 3. Results were too heterogeneous to allow meta-analysis.

8 studies correlated clinical DGE with gastric scintigraphy. Of these, 2 studies by Patti et al. and Hunt and Maclean, found no correlation between clinical DGE and scintigraphy [22, 23]. One study by Shan et al. [41] compared rates of clinical (subjective) DGE (‘sDGE’) and objective DGE (‘oDGE’). In the pylorus-preserving PD group, 42% had sDGE and 42% had oDGE, while in the pylorus-resecting PD group, 15% had sDGE and 88% had oDGE. However, no conclusion was drawn on the correlation between the clinical and objective DGE. Eshuis et al. [44] found a strong association between scintigraphy results and clinically relevant DGE (grade B or C) and concluded that gastric scintigraphy performed on day 7 following surgery predicted the severity of clinical DGE. Similarly, van Samkar et al. [43] found that scintigraphy performed at day 10 and day 21 had 100% positive predictive value and 100% specificity for clinical DGE.

For the acetaminophen/paracetamol group, 2 studies implied a correlation between the clinical and objective measure of DGE. Although no statistical analysis was performed, both studies found that patients with low serum acetaminophen/paracetamol also had concurrent clinical DGE, suggesting correlation [16, 51].

Krishna et al. [18] was the only study using fluoroscopy which implied an association with clinical DGE, with all 3 patients requiring reinsertion of NG tube demonstrating prolonged gastric Gastrografin® retention.

Only 1 study by Chijiiwa et al. [56] implied a correlation between clinical DGE and the ^13^C-acetate breath test. No statistical difference in both the incidence of clinical and objective DGE were found in the subgroups.

**Table 3:** Correlation between clinical and objective measures of DGE

Year	Author	Correlation between clinical and objective DGE
*Gastric scintigraphy*		
1987	Patti et al. [22]	•No correlation found between immediate postoperative clinical DGE and late objective DGE
1989	Hunt and McLean [23]	•No correlation between clinical DGE post-op with objective gastric emptying 3 months later
2005	Shan et al. [40]	•Somatostatin group: 9/11 had clinical DGE and 10/11 had objective DGE •Non-somatostatin group: 3/12 had clinical DGE and 1/12 had objective DGE
2005	Shan et al. [41]	•Post-op day 14, clinical: 42% PPPD and 15% PD had DGE •Post-op day 14, objective: 52% for solid gastric emptying and 76% for liquid gastric emptying in PPPD and 88% for solid gastric emptying and 91% for liquid gastric emptying in PD were delayed •Post-op 6 months, objective: 4.7% for solid gastric emptying and 4.7% for liquid gastric emptying in PPPD and 30% for solid gastric emptying and 37% for liquid gastric emptying in PD were delayed
2007	Shan et al. [42]	•The proximal to distal stomach radiation count ratio was statistically smaller for those with clinical DGE than those without clinical DGE (*p* = 0.025) •The authors concluded that scintigraphic proximal to distal radiation ratio is a useful method for assessing DGE
2013	van Samkar et al. [43]	•Patients with ISGPS grade B or C had higher median residual activity in the stomach at 120 min than patients with no DGE or ISGPS grade A (94% vs 39%, *p* = 0.004) •10/12 of those patients with ISGPS grade B or C had objective DGE •Objective DGE on post-op day 7 is predictive of severity of clinical DGE
2015	Eshuis et al. [44]	•9/19 with objective DGE also had clinical DGE •3/24 without objective DGE developed clinical DGE, all due to intra-abdominal complications •Authors concluded a strong association between gastric scintigraphy and clinical DGE
2017	Samaddar et al. [15]	•Objective DGE at post-op day 10 -Sensitivity- 61.53 -Specificity- 100% -PPV- 100% -NPV-61.53% •Objective DGE at post-op day 21 -Sensitivity- 38.46% -Specificity- 100% -PPV- 100% -NPV-50%
*Acetaminophen/paracetamol absorption Test*		
2005	Strommer et al. [51]	•6/9 with clinical DGE also had objective DGE (1/9 later excluded) •12/22 without clinical DGE did not have objective DGE (2/22 later excluded) •Authors concluded that clinical DGE was associated with reduced objective gastric emptying
2014	Tamandl et al. [16]	•At any time point after ingestion of the test meal, the serum acetaminophen/paracetamol levels were lower in patients with DGE
*Fluoroscopy*		
2015	Krishna et al. [18]	•3/3 patients with clinical DGE also had prolonged retention of Gastrografin®
^*13*^ *C-acetate breath test*		
2009	Chijiiwa et al. [56]	•No significant difference in the incidence of clinical DGE (*p* = 0.34) and no significant difference in ^13^C-acetate breath test results between the antecolic and retrocolic groups

*DGE* delayed gastric emptying, *ISGPS* International Study Group in Pancreatic Surgery, *NPV* negative predictive value, *PD* pancreaticoduodenectomy, *PPPD* pylorus-preserving pancreaticoduodenectomy, *PPV* positive predictive value

### Correlations between symptoms and objective DGE

3 objective measures of DGE had implied or explicit correlations between symptoms (including abdominal pain, early satiety, nausea and vomiting and/or loss of appetite) and objective DGE, with 1 study each (Table 4). No studies were identified for fluoroscopy.

For gastric scintigraphy, Pastorino et al. [30] demonstrated a reduced gastric emptying time (49.3 min compared to 82.3 min) with patients who had better clinical outcomes (score 1 and 2 according to the questionnaire used) compared to those with a poorer clinical outcome.

For the acetaminophen/paracetamol absorption test, Takeda et al. [49] found that the improvement of test results coincided with the recovery of symptoms. Specifically, the area under the curve at 90 min following administration was 48.1% at 1 month postoperatively which returned to preoperative baseline values at 6 months, coinciding with the improvement in symptoms.

For ^13^C-acetate breath test, only 1 study by Kawai et al. [20] demonstrated symptoms in the immediate postoperative period was associated with DGE on the ^13^C-acetate breath test months after the surgery, suggesting association.

**Table 4:** Correlation between objective measures of DGE and patient symptoms

Year	Author	Correlation between symptoms and objective DGE
*Gastric scintigraphy*		
1995	Pastorino et al. [30]	•Patients with a better clinical outcome score of 1 and 2 was associated with improved gastric emptying half-life time compared to worse clinical outcome scores of 3 and 4 (49.3 vs 82.3 min average)
*Acetaminophen/paracetamol absorption Test*		
1999	Takeda et al. [49]	•The recovery of gastric emptying time was associated with improvement in patient symptoms
^*13*^ *C-acetate breath test*		
2014	Kawai et al. [20]	•Patients without objective DGE had improved dietary intake long-term and recovery of body weight

*DGE* delayed gastric emptying

## Discussion

Delayed gastric emptying is a common complication following PD and is associated with increased morbidity, prolonged hospitalization and increased costs to healthcare [3, 4]. While the ISGPS definition for DGE is the most widely used, it relies on subjective clinical judgement and only diagnoses DGE at the end of the clinical course. This literature review identified 4 objective modalities to assess the presence of DGE following PD. These include gastric scintigraphy, acetaminophen/paracetamol absorption test, fluoroscopy and ^13^C-acetate breath test. All modalities had at least 1 study that explicitly or implied a correlation with clinical DGE definition.

Historically, DGE assessments and definitions have been heterogeneous, making meaningful comparisons between different studies difficult. However, in 2007, the ISGPS developed a consensus definition for DGE following pancreatic surgery [4]. This definition classified the severity of DGE (A, B or C) based on the duration of NG intubation or reinsertion, with the condition of no underlying mechanical obstruction to cause symptoms. Only 1 study in this review clearly included this condition in their methodology [13]. Moreover, DGE can be further subclassed into primary or secondary, dependent on the presumed cause being attributed to the surgical procedure or postoperative complications, respectively [7]. While secondary DGE is expected to resolve following the treatment of postoperative complications, this is not necessarily true for primary DGE, and is therefore the focus of this review.

Several studies have now validated the ISGPS definition. These studies have found statistically significant differences in postoperative clinical outcomes, including further diagnostic evaluations (such as endoscopy or imaging), treatment, parenteral nutrition, ICU admission duration and overall hospitalization duration between the different DGE severities (including those with no DGE) [1, 5, 6]. Since the consensus statement, almost all studies examined in this literature review, including those exclusively using the clinical definition, used the ISGPS definition.

There are several advantages to the clinical ISGPS definition of DGE. In particular, it is non-invasive and requires minimal cost to the patient or health system. It allows a standardized definition for audit and research purposes and the development of risk stratification tools for DGE following PD [63]. However, there are some disadvantages. Firstly, this definition relies on clinician judgement on whether to maintain or re-insert the NG tube. This decision is based on symptoms (e.g., food intolerance, nausea, vomiting), which, albeit pragmatic, is an experience-based assessment of the patient’s underlying gastric physiological status. Secondly, medications, such as anti-emetics or prokinetics, may also influence patient symptoms and therefore clinician judgement [46]. Finally, the ISGPS definition is a retrospective assessment and while it is useful for audits and research, it does not allow a real-time diagnosis of DGE and thereby does not aid in guiding immediate inpatient management or predicting recovery. As such, several authors have proposed that a real-time and objective measure of DGE may provide a more accurate way of assessing the patient’s true gastric physiology, to potentially allow future research into this area to become more standardized and therefore guide postoperative management, such as decision for early parenteral nutrition support if stomach recovery is expected to be prolonged or other novel therapies [15, 39, 41, 43, 64]. Indeed, no studies identified in this review used the results of the objective assessment of DGE to guide inpatient management, thus would be a focus for future research. An ideal test would not only exclude mechanical obstruction but also assesses gastric motility, and there is currently a lack of such testing modality.

This literature review has identified several non-clinical or objective measures to assess and define DGE with the most common technique being gastric scintigraphy. First described by Griffith et al. [65] in 1966, this technique is still considered the standard for objectively assessing gastric emptying [58, 66], with a 2008 consensus statement developed by Abell et al. [58] to standardize protocols. Limitation to this measure include its relative cost, access to equipment, impracticality to apply in the immediate postoperative setting and concerns on the use of radioactive isotopes [46, 50]. Moreover, it may also not be feasible to administer the test meal in patients experiencing severe nausea or vomiting. Eight studies assessed the presence of associations between clinical DGE and gastric scintigraphy. Three studies, all published prior to 2006, either found no correlation between clinical DGE and gastric scintigraphy or did not conclude a correlation between the two measures. In contrast, the more recent studies have all found or implied a correlation between clinical DGE definitions and gastric scintigraphy [15, 40–44], which may be due to the standardization of DGE assessment protocols and definitions.

The other non-clinical or objective measures of DGE identified in the literature review all have advantages and disadvantages. In the acetaminophen/paracetamol absorption test, the advantages include its relative accessibility, the benefits of a bedside test, that it does not involve the nuclear medicine department and avoids radiation. However, it does require serial blood tests posing risks and requiring intensive input by both clinicians and laboratory. It is also not a direct measure of gastric emptying, albeit being correlated with clinical DGE following PD [58]. In the ^13^C-acetate breath test, the main advantage is its non-invasive nature. However, there are concerns of unreliable results following pancreatic surgery due to the possibility of altered physiology and intestinal absorption, thereby affecting test accuracy and reliability [67]. For fluoroscopy, the main advantages are that this technique is well-established in other fields of medicine, is readily available and allows a real-time assessment of gastric function. Limitations include its subjective nature, difficulty in quantifying results and radiation exposure.

While the aforementioned objective measures of gastric emptying are valuable measures of gastric function, they all represent indirect *functional* measures of gastric transit in contributing to the patient’s symptoms. Rather, it may be more useful to assess the direct *physiological* status of gastric motility after pancreatoduodenectomy, particularly in patients with primary DGE to inform targeted treatments. By understanding the underlying pathophysiology of these patients, rather than functional status, clinicians may potentially be able to predict, assess and even aim to treat DGE with novel strategies, such as gastric pacing or ablation, which are currently under research [68]. Numerous studies have investigated gastric physiology or function by assessing either the peristaltic or electrophysiological activity of the stomach, termed gastric slow waves [69, 70]. Non-surgical and post-surgical gastric dysfunctions have been associated with gastric slow wave abnormalities [71–73]. Recent bioengineering developments now allow for more accurate quantification of these gastric slow waves to be possible, particularly with the recent development of non-invasive high-resolution electrogastrography devices to assess gastric electrical activity [68]. This validated technique has been correlated with patient symptom severity in other conditions, albeit never in PD patients [74]. Further research is now required to determine whether these novel techniques may better assess the pathophysiology of DGE post-PD [35, 75].

The strength of this review was the broad search strategy, which allowed a large number of studies to be included in this literature search. The main limitation of this review was the relatively few studies for each objective technique with heterogeneous protocols (including different interventions and time points after surgery), meaning that statistical comparisons between various techniques (e.g. network meta-analysis) could not be performed. A further limitation was the lack of high-quality studies and studies directly comparing objective assessment of DGE with clinical DGE or symptoms. This review now provides the foundations for future research into this area of clinical assessment and the development of an objective clinical tool to more accurately assess DGE following PD.

## Conclusion

This literature review identified several techniques which objectively assess gastric function following surgery, with the most common being gastric scintigraphy. There is currently no consensus on the preferred objective measure of delayed gastric emptying following pancreatic surgery. Therefore, a consensus may be useful in defining or developing a more objective and standardized measure of delayed gastric emptying following pancreaticoduodenectomy.
